# The utilization of a magneto-fluorescence sequential labeling strategy for the visualization and tracking of intestinal translocation of oral microbiota

**DOI:** 10.1016/j.mtbio.2026.103062

**Published:** 2026-03-27

**Authors:** Meiling Jing, Xiuli Wang, Yaoxia Li, Leyi He, Wenduo Tan, Yujie Zhang, Yueyi Yang, Jing Huang, Zhengwei Huang, Changchun Wang, Chenguang Niu

**Affiliations:** a Department of Endodontics, Shanghai Ninth People's Hospital, Shanghai Jiao Tong University School of Medicine, College of Stomatology, Shanghai Jiao Tong University, Shanghai, China; b National Clinical Research Center for Oral Diseases, National Center for Stomatology, Shanghai Key Laboratory of Stomatology, Shanghai, 200011, China; c State Key Laboratory of Molecular Engineering of Polymers, Department of Macromolecular Science, and Laboratory of Advanced Materials, Fudan University, Shanghai, 200433, China

**Keywords:** Magnetic supraparticle, D-amino acid derivatives, Oral-gut axis, Microbiota, Bioimaging, Metabolic labeling

## Abstract

The microbiota of the oral-gut axis influences systemic physical health, but the exact mechanisms remain unknown. In this study, magnetic supraparticles modified with polyacrylic acid and 3-azido-D-alanine hydrochloride (MSP@PAA-ADA) were constructed, and a magneto-fluorescence sequential labeling strategy was established for microbiota viability labeling in combination with a cyanine 5-labeled D-amino acid (Cy5ADA) fluorescent probe. MSP@PAA-ADA, with a particle size of approximately 160 nm, exhibited good hydrophilicity, high magnetic responsiveness, and low bacterial toxicity. MSP@PAA-ADA achieved efficient labeling of oral bacteria through interaction with peptidoglycan in bacterial cell walls. The experimental results indicated that MSP@PAA-ADA can be able to widely bind salivary microbiota, and the labeled salivary bacteria translocated along the oral-gut axis. A small amount of fluorescent signal could still be detected in the mice 36 h after gavage. MSP@PAA-ADA was employed as a magnetic probe for the *in vitro* labeling of oral bacteria, and sequential labeling was conducted using the fluorescent metabolic probe Cy5ADA. The oral bacteria labeled with MSP@PAA-ADA can be effectively magnetically separated after intestinal translocation through the gastrointestinal tract. The magnetically responsive and fluorescently imaged oral bacteria demonstrate their activity after intestinal translocation. The magneto-fluorescence sequential labeling strategy that enables controllable spatiotemporal tracing and simultaneous viability assessment of intestinal oral bacteria in the gut, providing a new technical approach for dissecting biological mechanisms along the oral–gut microbiota axis.

## Highlights

• MSP@PAA-ADA enables efficient labeling of salivary bacteria without affecting viability.
• Co-labeling of MSP@PAA-ADA and Cy5ADA reveals salivary microbiota intestinal translocation.
• Magneto-fluorescence tracking reveals transient bioactivity of oral bacteria in gut.
• The strategy enables real-time longitudinal tracking for oral-gut axis microbiota research.

## Graphical abstract

**Figure:**
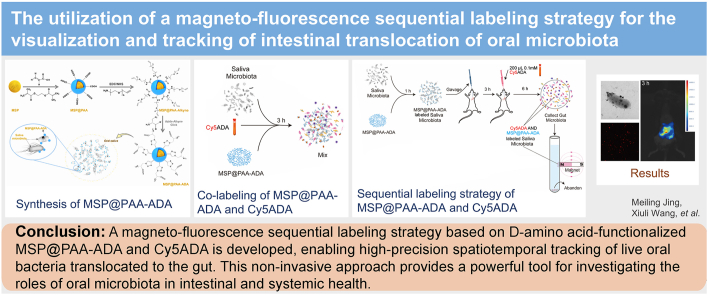

## 1 Introduction

The human body harbors complex and dense microbial communities, with the oral cavity and the gut being the two most densely populated sites. The interaction between these sites forms the oral-gut microbiome axis. While the oral cavity is constantly exposed to external stimuli that shape subsequent microbial communities, the presence of strong physicochemical and immune barriers in the digestive tract limits the transfer of most microbes [1,2]. Nevertheless, numerous studies have identified a correlation between dysbiosis in the oral-gut axis and a variety of diseases, including inflammatory bowel disease, colorectal cancer, and esophageal cancer [3]. However, the precise biological mechanisms by which oral microbes survive in this new ecological niche of the gut and influence systemic health remain incompletely understood [4].

Current research into the oral-gut axis primarily relies on high-throughput gene sequencing technologies. Although conventional sequencing can detect microbial presence and abundance, it fundamentally lacks the capacity to differentiate between metabolically active live colonization and mere residual DNA [5,6]. This limitation significantly hinders the real-time, dynamic observation of the oral microbiota's ectopic colonization process within the gut. Furthermore, alternative methods like isotope labeling are often prohibitively costly and lack the required spatial resolution [7]. To address this, Fluorescent D-amino acid (FDAA) probes utilize bacterial transpeptidase activity to incorporate non-natural D-amino acids into newly synthesized cell wall peptidoglycan [8,9]. This labeling strategy has been demonstrated as a powerful tool for visualizing and distinguishing viable, metabolically active bacteria [10]. In recent years, a range of Cy5ADA-based imaging strategies have been developed, all of which utilize stepwise labelling with multiple fluorescent probes to visualize distinct gut microbes [11]. However, the isolation and subsequent collection of these microbial communities for further research remains a challenging task.

In this study, we successfully designed and prepared amino-D-alanine (ADA)-modified magnetic composite microspheres (MSP@PAA-ADA). We utilized these microspheres as a magnetic solid-phase adsorption unit and a dual-function targeting/metabolic probe for oral bacteria, which, combined with fluorescent labeling technology, established a novel strategy for detecting the intestinal translocation of oral bacteria along the intestinal axis. Ultimately, our work provides a powerful new tool for deepening the mechanistic understanding of the *in vivo* behavior and pathogenic roles of the oral-gut axis microbiota.

## 2 Materials and methods

### 2.1 Materials

Acrylic acid (AA, 99%), 1-ethyl-3-(3-dimethylaminopropyl) carbodiimide (EDC), n-hydroxysuccinimide (NHS), 2,2-azobis(isobutyronitrile) (AIBN, 98%) and copper(I) bromide (CuBr, 99%) were obtained from Shanghai Aladdin Chemistry Co., Ltd. Anhydrous ferric chloride (FeCl_3_, 97%), poly (4-styrenesulfonic acid-co-maleic acid) sodium salt (PSSMA, average Mw ≈ 20,000, SS/MA = 1:1), ethylene glycol (EG, 99%) and sodium hydroxide (NaOH, ≥96%) were obtained from Sinopharm Chemical Reagent Co., Ltd. Anhydrous sodium acetate (NaAc, 99%), acetonitrile (AN) and ethylene glycol dimethacrylate (EGDMA) were purchased from Shanghai Macklin Biochemical Co., Ltd. LIVE/DEAD viability/cytotoxicity assay kit and phosphate buffer (PBS, pH 7.4) were synthesized by Sangon Biotech Co., Ltd. (Shanghai, China). 3-azido-D-alanine hydrochloride (ADA) and D-amino acids modified with the near-infrared fluorescent dye Cyanine 5 (Cy5ADA) were all purchased from Qiangyao Biotechnology Co., Ltd. Gentian violet staining solution, iodine solution, and safranine solution were purchased from Zhuhai Bioso Biotechnology Co., Ltd. Four-week-old C57BL/6J mice were obtained from Suzhou Cyagen Biosciences Co., Ltd. Amino-polyethylene glycol-alkyne (NH_2_-PEG-Alkyne, Mw ≈ 1000) was purchased from Shanghai Tuoyang Biotechnology Co., Ltd. SDS-PAGE gel rapid preparation kit was obtained from Shanghai Beyotime Biotechnology Co., Ltd.

### 2.2 Synthesis of magnetic supraparticle (MSP)

The synthesis of magnetic supraparticle (MSP) was carried out using microwave method. Typically, FeCl_3_ (1.95 g), NaAc (9.02 g), PSSMA (4.5 g), and NaOH (1.8 g) were dissolved in EG (60 mL). The resulting mixture was stirred at 70 °C for 1 h to form a homogeneous solution and then transferred into a reaction vessel of the microwave reactor. The reaction was conducted under the following conditions: a temperature of 200 °C, microwave power of 160 W, heating time of 1 min, and reaction time of 20 min, using a single-stage heating mode. Upon completion of the reaction, the obtained black product was washed with water and ethanol for several times with the help of a magnet and eventually dispersed in water for further use.

### 2.3 Synthesis of MSP@PAA microspheres

Using MSP as the magnetic core, the shell of the microsphere was prepared by reflux precipitation polymerization of AA (poly (acrylic acid) (PAA)), with EGDMA as the cross-linker, and the final core-shell microspheres were abbreviated as MSP@PAA. Typically, MSP (50 mg), AA (100 mg), EGDMA (200 mg), and AIBN (6 mg) were dispersed in AN (40 mL) and sonicated for 3 min to form homogeneous solution. The reaction was carried out at 95 °C for 90 min under reflux. The MSP@PAA microspheres were collected by a magnet and washed with water and ethanol several times to remove the excess reactants and byproducts.

### 2.4 Synthesis of MSP@PAA-Alkyne microspheres

Grafting PEG molecules onto MSP@PAA microspheres were executed through EDC/NHS coupling method between the carboxyl group of PAA and the amino group of NH_2_-PEG-Alkyne. Specifically, the MSP@PAA microspheres (35 mg) were dispersed in AN (20 mL) by ultrasonication. EDC (48 mg) and NHS (48 mg) were added sequentially to the mixture, which was then stirred continuously at 70 °C for 60 min. Subsequently, NH_2_-PEG-Alkyne (500 mg) was added all at once, followed by stirring continuously overnight at 70 °C. The product was washed with water and ethanol for several times with the help of a magnet and eventually lyophilized for further use.

### 2.5 Synthesis of MSP@PAA-ADA microspheres

The ADA molecules were attached to the microspheres via click chemistry between the alkyne groups on the surface of MSP@PAA-Alkyne microspheres and the azide groups of ADA. Typically, the MSP@PAA-Alkyne (50 mg) microspheres were ultrasonically dispersed in DMF (50 mL), followed by the sequential addition of ADA (150 mg) and CuBr (50 mg). The mixture was then stirred at room temperature overnight. The resulting product was magnetically separated, washed repeatedly with 75% ethanol, and then dried to constant weight for further use.

### 2.6 Collection of oral saliva bacteria

Saliva samples were collected according to Navazesh (1993) criteria. 30 min prior to collection, subjects rinsed their mouth thoroughly with purified water and then sat comfortably. 5 min prior to collection, subjects tilted their head slightly to minimize oral and facial movements. During sampling, subjects were required to accumulate saliva at the bottom of their mouth and pour saliva into sterile tubes at 60 s intervals. Take 5 mL saliva sample into sterile centrifuge tube, sieve the microbiota in 70 μm cell sieve to remove tissue debris in saliva. Centrifuge the sieved saliva at 3000 rpm for 10 min, aspirate the supernatant, take the precipitate into sterile tube, resuspend it with PBS buffer solution (1 × , pH = 7.4), and then dispense it into sterile tube in equal volume. The bacterial count in each tube is about 1 × 10^9^ CFU, so as to obtain the sample solution containing oral saliva bacteria.

### 2.7 *In vitro* labeling of microbiota

The magnetic composite microspheres were weighed and added into the above oral saliva bacterial sample solution, and the final concentration of magnetic microspheres was 0.1 mg/mL, immediately vortexed and mixed, and then incubated in a constant temperature oscillator at 37 °C and 80 rpm for 1 h. The incubated sample was slowly added into sterile PBS, and then magnetic separation was performed by using magnets. Disperse the collected sample product in 1x PBS, centrifuge at 3000 g for 5 min, collect the precipitate, and resuspend it in 1x PBS. Repeat the process three times to obtain the magnetic composite microspheres-labeled oral saliva bacterial sample.

### 2.8 Bacterial toxicity test

*Fusobacterium nucleatum* (*F. n*) was used as an oral model bacterium, and bacterial culture solutions containing different amounts of magnetic composite microspheres were cultured for proliferation. Specifically, *F*. *n* was inoculated into BHI liquid medium and cultured anaerobic overnight at 37 °C. The bacterial solution cultured overnight was inoculated into BHI liquid medium at a ratio of 1:10 and cultured anaerobic at 37 °C for 2 h. 2.6 mg/mL MSP@PAA-ADA dispersion solution was prepared and sterilized. In the biosafety cabinet, take a sterile 96-well plate, add 200 μL of MSP@PAA-ADA dispersion solution to the first three rows of the first column, add 100 μL of sterile PBS solution to the remaining wells, dilute backwards from the first column, and transfer 100 μL of liquid to the next well for two-fold dilution until the 11th column. Add 100 μL PBS as negative control in the 12th column. Then, add 100 μL of diluted bacterial solution to each well, and incubate anaerobic at 37 °C overnight. 100 μL of bacterial solution cultured overnight was aspirated from each well plate, diluted 10^1^-10^8^ times with PBS by gradient dilution method, 50 μL of each diluted bacterial solution was evenly spread on brain heart extract broth solid medium (BHI), incubated anaerobic overnight at 37 °C. The colonies were counted according to the number of mono-clone bacteria on the culture dish and corresponding dilution times, and the relative survival rate of bacteria was calculated. To ensure the accuracy of the test, at least 3 parallel experiments were performed for each well sample, and statistical analysis was performed using *t*-test with 95% confidence (*p* < 0.05).

Further, oral saliva samples before and after labeling with magnetic composite microspheres were subjected to bacterial live-death test to evaluate the bacterial toxicity of the material. Fluorescence staining of sample bacteria was performed according to the operation procedure of LIVE/DEAD kit instructions, and samples were sampled and analyzed on confocal fluorescence microscope. SYTO9 fluorescent dye was used to label bacteria with intact cell membrane, and it showed green color at excitation wavelength of 480 nm. Propidium iodide (PI) labeled bacteria with damaged cell membranes appear red at 490 nm excitation wavelength.

### 2.9 Gram staining

The bacteria collected were spread evenly on a coverslip and the bacterial solution was dried close to an alcohol lamp for seconds. The gram staining was performed using Gram staining kit (Baso, China) according to the instruction manual. Then samples were visualized by optical microscope (Zeiss, German).

### 2.10 16S rRNA sequencing

The QIAamp DNA Mini Kit (Qiagen, Valencia, CA, USA) was used to extract total bacterial DNA. NanoDrop 2000 UV-vis spectrophotometer (Thermo Scientific, Wilmington, DE, USA) and 1% agarose gel electrophoresis were used to quantified DNA. The 16S rRNA genes was amplified using the following primers: 338F forward primer (5′-ACTCCTACGGGAGGCAGCAG-3′) and 806R reverse primer (5′-GGACTACHVGGGTWTCTAAT-3′) by thermocycler PCR system (GeneAmp 9700; Applied Biosystems, Carlsbad, CA, USA) as described previously [12]. The purified amplicons were sequenced using the Illumina Miseq PE300 platform (Illumina, San Diego, CA, USA) according to the standard protocol. Operational taxonomic units (OTUs) were clustered with 97% similarity using UPARSE (version 7.1, http://drive5.com/uparse/) and matched to a database (SILVA 106; https://www.arb-silva.de/) for taxonomic analysis [13,14]. A modified OTUs table was generated for subsequent analysis after subsampling according to the minimum number of sample sequence. Alpha diversity indexes (Ace, Chao, Shannon and Simpson) and co-occurrence networks of the 50 most abundant genera of each group were calculated to evaluate the diversity and construction of community species using MOTHUR (version 1.30.2, https://www.mothur.org/wiki/Download_mothur) [15]. Community barplot analysis and heatmaps on genus level were created via R platform (version 3.6.1) to describe the species composition in different groups. The weighted UniFrac principal coordinates analysis (PCoA) and partial least squares discriminant analysis (PLS-DA) were performed to evaluate the variances of sample community composition using QIIME [16,17]. The linear discriminant analysis (LDA) effect size analysis (LEfSe, http://huttenhower.sph.harvard.edu/galaxy) was applied to identify the most discriminatory taxa among groups from phylum to genus level with logarithmic LDA score >2.0 regarded as discriminative species [18,19]. The unweighted Unifrac distance-based redundancy analysis (db-RDA) and Spearman's rank correlation coefficient were operated to quantitatively evaluate the multicollinearity relationship between environmental/clinical factors and sample species composition [20]. Phylogenetic Investigation of Communities by Reconstruction of Unobserved States (PICRUSt2, version 1.1.0, http://picrust.github.io/picrust/) analysis and Wilcoxon rank sum test with a Benjamini–Hochberg false discovery rate (FDR) correction to adjust *p* values for multiple testing were performed to predict and compare the abundance of Kyoto Encyclopedia of Genes and Genomes (KEGG) pathways in different groups.

### 2.11 Acquisition of magneto-fluorescence dual-labeled oral saliva samples

Add 0.1 mg/mL MSP@PAA-ADA and 0.1 mM Cy5ADA to the standard saliva sample solution, vortex and mix well, and incubate at 80 rpm for 1 h at 37 °C, protected from light, and then centrifuge the incubated sample mixture, and resuspend the upper layer of the turbid liquid in sterile PBS after discarding the turbid liquid, and then perform the magnetic separation and resuspend it in sterile PBS after being washed with PBS buffer. The process was repeated twice to obtain oral saliva samples double-labeled with magnetic composite microspheres and fluorescent probes.

### 2.12 *In vitro* co-labeling feasibility assessment

Gram staining and laser confocal imaging of dual-labeled oral salivary bacteria were performed to assess the feasibility of dual-probe co-labeling of microbiota. The experimental procedure for Gram staining is described in 2.9. Laser confocal imaging was performed as follows: the colony samples obtained by magnetic separation were inoculated into confocal cell culture dishes (d = 35 mm) covered with SDS-PAGE protein gels. The samples were excited with a laser at a wavelength of 638 nm, and fluorescent images of the bacterial cells were obtained by detecting and capturing the emitted light waves using the corresponding emission filters.

### 2.13 *In vivo* imaging experiment

The c57/BL6J mice were kept in an independent ventilated cage with a cycle of 12 h light and 12 h dark. All mice were fed for one week after purchase to adapt to the environment. Gavage 200 μL of the obtained bacterial solution to mice that have fasted overnight in advance. After the mice were allowed to stand for 3, 9, 16, 24 and 36 h, they were pre-anesthetized using 3% isoflurane. Then, the mice which had their abdomens depilated were transferred to the VISQUE Invivo Smart-LF device and maintained at a flow rate of 1 L/min and 2% isoflurane for continuous anesthesia. The images were captured and analyzed in real time by CleVue 3.1.3.2054 software according to the instruction manual.

The anesthetized mice were euthanized and the gastrointestinal tract was immediately removed to obtain *ex vivo* fluorescence imaging images of the digestive tract under this system.

### 2.14 Transmission electron microscope (TEM)

The sample should be deposited onto the sample plate, the front of the copper mesh attached to the surface of the sample, and the mesh removed after a period of 3 min. The resultant sample should then be left to dry at room temperature. The 2% phosphotungstic acid dye solution should be applied for a period of 1-2 min. The copper mesh should then be removed, and the excess dye solution should be absorbed using filter paper. The copper mesh was observed using a JEM-1400FLASH TEM (Japan Electronics Corporation). The mesh was initially observed at low magnification, and the area to be examined was selected for image capture.

### 2.15 Characterizations

Dynamic light scattering instrument (DLS, Malvern ZEN3600) was used to record the hydrodynamic particle size, distribution of microspheres and zeta potentials under a helium-neon laser light source. Transmission electron microscopy (TEM, Tecnai G2 20 TWIN) was used to characterized microspheres’ size at an accelerating voltage of 200 kV. Scanning Electron Microscopy images (SEM, Hitachi S-4800) were obtained using a scanning electron microscope at an accelerating voltage of 20 kV. Fourier transform-infrared spectra (FT-IR, Nicolet NEXUS-470) were determined in the 400-4000 cm^−1^ range by using pressed KBr pellets. Thermogravimetric analysis measurements (TGA, PerkinElmer) were taken on a Pyris 1 TGA instrument, and the temperature was first increased from room temperature to 100 °C, held until a constant weight, and then increased from 100 to 800 °C at a rate of 10 °C/min. Magnetization curves were acquired using a model 6000 magnetometer (Quantum, USA) at 300 K. The Primo Star optical microscope from ZEISS was used to visualize the microbiota after Gram staining. The Vieworks Smart-LF mouse *in vivo* imager was used to capture images of salivary microbiota after gavage of mice for different time periods, and to trace their distribution in the body to characterize their translocation in the body. The NIR excitation wavelength was set to 638 nm and systematic analysis was performed using CleVue software, and the acquired images were further processed on Living Image software to deduct the interference of background autofluorescence. The ultra-high-resolution point-and-shoot laser confocal microscope model LSM880 from ZEISS was used for excitation and fluorescence imaging of the microbiota samples for capturing.

### 2.16 Statistical analysis

Statistical analyses were performed with the GraphPad Prism 9.0.0.121 software (GraphPad, USA) and are presented as means ± standard deviations (SD). The normal distribution of the data was confirmed with Q–Q plots. All data were analyzed statistically with *t*-test or Ordinary one-way ANOVA. For all statistical tests, data were provided as means ± SD. *p* < 0.05 was considered statistically significant.

## 3 Results

### 3.1 Preparation and characterization of MSP@PAA-ADA

**Fig. 1:**
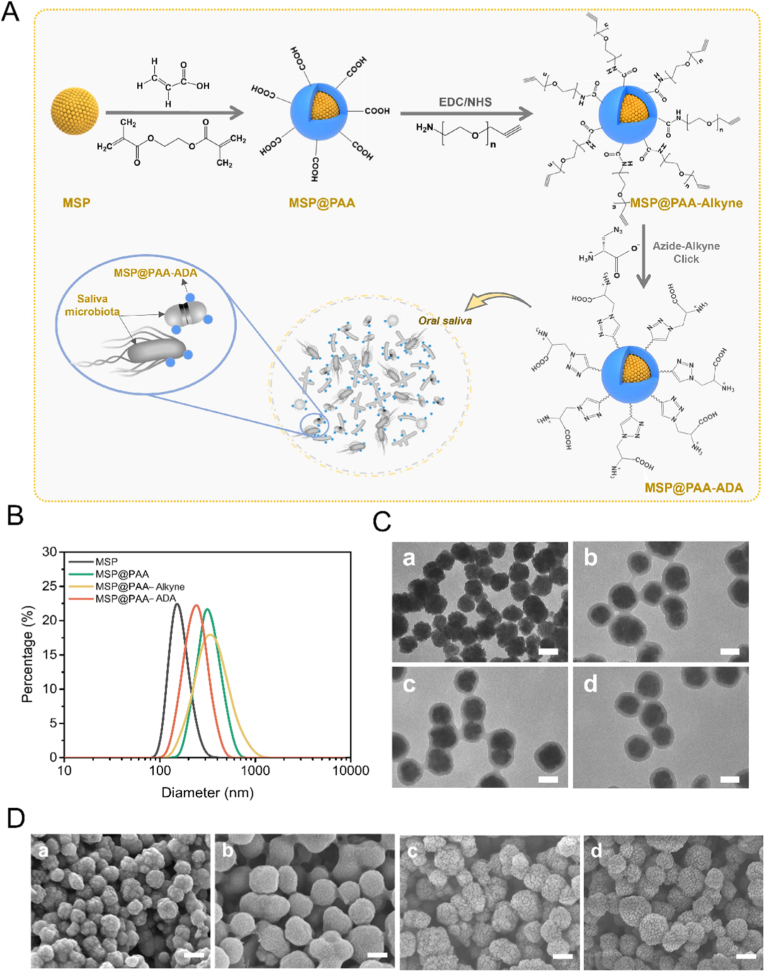
The structural selection and physical property testing of microspheres. (A) Flowchart for the synthesis process of MSP@PAA-ADA microspheres. (B) Size distributions of MSP, MSP@PAA, MSP@PAA-Alkyne, and MSP@PAA-ADA. (C) TEM images of MSP (a), MSP@PAA (b), MSP@PAA-Alkyne (c), and MSP@PAA-ADA (d). (D) SEM of MSP (a), MSP@PAA (b), MSP@PAA-Alkyne (c), and MSP@PAA-ADA (d). Scale bar is 100 nm.

The protocol for the synthesis of MSP@PAA-ADA is illustrated in Fig. 1A. Briefly, MSP of Fe_3_O_4_ nanocluster stabilized by PSSMA were first synthesized. The crosslinked PAA layer was then coated on the surface of MSP through reflux precipitation polymerization. Next, the PEG spacers containing alkyne groups were modified on the surface of the microspheres through the amidation reaction between the amine groups of NH_2_-PEG-alkyne and the carboxyl groups on the surface of the microspheres, which is conducive to better hydrophilic stability of the composite microspheres. The presence of alkyne in the NH_2_-PEG-alkyne moiety enables efficient immobilization of ADA on the microsphere surface through click chemistry reaction between ADA and alkyne.

The MSP had an average diameter of approximately 100 nm. After encapsulation with cross-linked PAA, the size of the composite microspheres increased to around 160 nm (Fig. 1B and S1), and they possessed a well-defined core-shell structure (Fig. 1C). The morphology of the microspheres did not change significantly after stepwise modification with NH_2_-PEG-Alkyne and ADA (Fig. 1C). Furthermore, the hydrodynamic diameter (D_h_) of microspheres indicated that MSP@PAA had a larger D_h_ compared to MSP, which could be attributed to the increased size of the microspheres due to the coating shell as well as the enhanced hydrophilicity of their surfaces resulting in a hydration layer. The modification with NH_2_-PEG-Alkyne lead to a slightly increased D_h_ for MSP@PAA-Alkyne, whereas the size distribution of MSP@PAA-ADA became significantly narrower after further modification with ADA, with the dispersity index of approximately 0.091, indicating that MSP@PAA-ADA had better dispersion stability in water (Fig. 1B). SEM was employed to characterize the nanostructures of obtained magnetic microspheres. MSP was composed of aggregated nanocrystalline clusters with a rough surface (Fig. 1D). After being coated with a polymer shell, MSP@PAA exhibited a smoother surface as well as significantly larger size compared to MSP (Fig. 1D). Furthermore, the surface of the composite microspheres became gradually rougher again after stepwise modification with NH_2_-PEG-Alkyne and ADA (Fig. 1D).

**Fig. 2:**
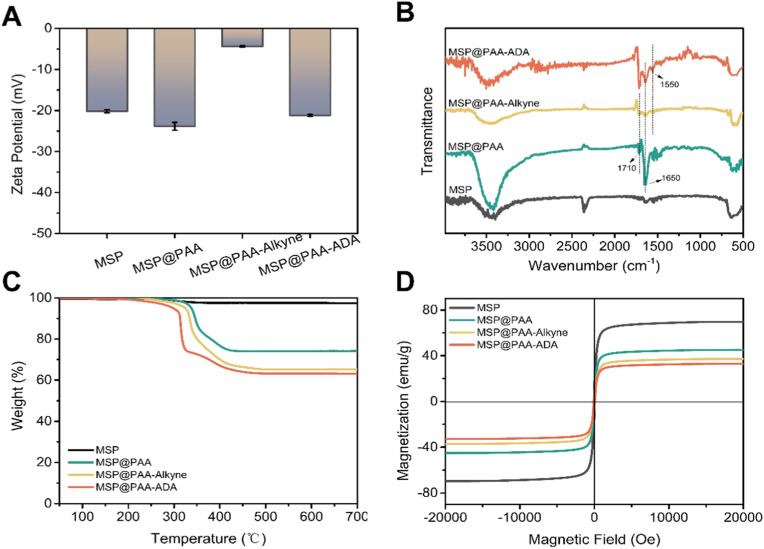
Characterization of the functional microspheres. (A) Zeta potential, (B) FT-IR spectra, (C) TGA curves, and (D) magnetic hysteresis curves of MSP, MSP@PAA, MSP@PAA-Alkyne, and MSP@PAA-ADA.

**Fig. 3:**
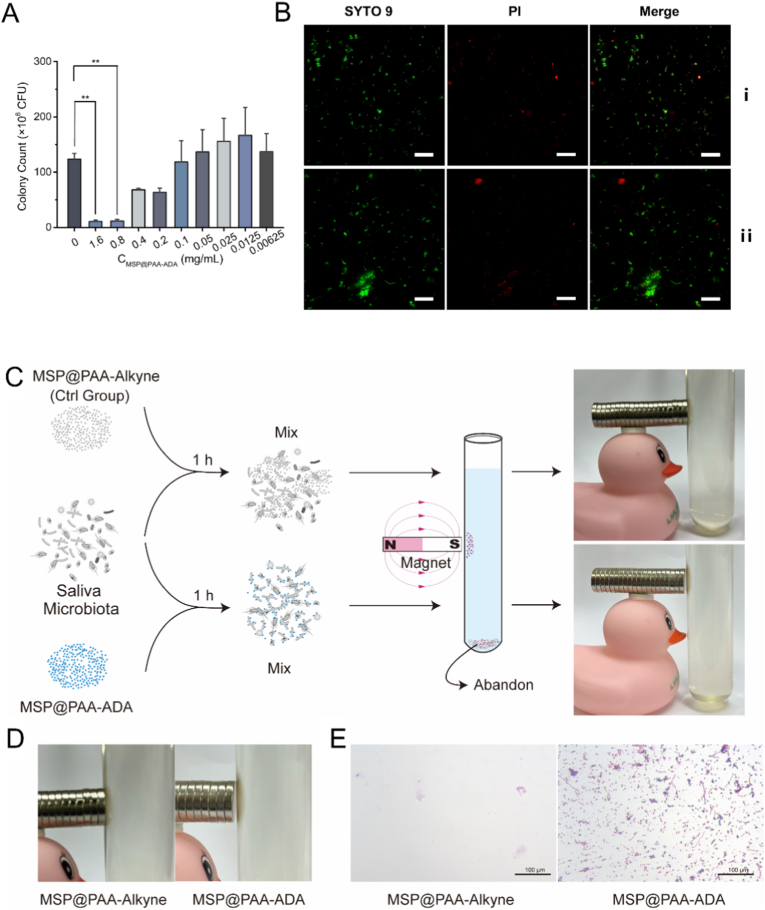
The performance of MSP@PAA-ADA in labeling bacteria *in vitro*. (A) Proliferations of *F n* cultured with different concentrations of MSP@PAA-ADA, detected by single colony counting. (B) Live bacteria (SYTO 9, Green) and dead bacteria (PI, Red) of salivary microbiota before (i) and after (ii) co-culturing with 0.1 mg/mL MSP@PAA-ADA for 1 h. Scale bar is 50 μm. (C) Schematic illustration of the magnetic detection process. (D) A magnified view showing the material adsorbed around the magnet (E) The Gram stanning of salivary microbiota cultured with MSP@PAA-Alkyne and MSP@PAA-ADA after separation magnetic separation. Scale bar is 100 μm. (For interpretation of the references to color in this figure legend, the reader is referred to the Web version of this article.)

As visualized in Fig. 2A, the magnetic composite microspheres exhibited negative surface charge. After coating with a carboxylated polymer, the zeta potential of MSP@PAA became more negative, slightly increasing from the average potential of MSP (−20 mV) to −24 mV. Subsequently, the stepwise modification with NH_2_-PEG-Alkyne and ADA enabled the zeta potential of the composite microspheres to first drop significantly and then increase substantially. The zeta potential of the final MSP@PAA-ADA microspheres after ADA modification was about −21 mV. The chemical composition of microspheres was characterized by FT-IR. As displayed in Fig. 2B, the FT-IR spectrum of MSP exhibited the characteristic peaks of the Fe-O vibration at 588 and 633 cm^−1^. Compared to MSP, the new absorption peaks appeared at 1720 and 1650 cm^−1^ in the MSP@PAA, which were attributed to the C=O stretching vibration of ester groups in EGDMA and the C=O stretching vibration of carboxyl in AA, respectively. And there were an increase in peak intensity at these two positions after grafting NH_2_-PEG-Alkyne with the PEG spacer chain onto the surface of composite microspheres. For the sample of MSP@PAA-ADA, the modification with ADA lead to increased peak intensities at 1650 and 1550 cm^−1^, which could be respectively ascribed to the C=O stretching vibration of carboxyl and N=N stretching vibrations of azide groups in ADA. Additionally, the new absorption peak at 1363 cm^−1^ was allowed for the N-N bond formed by the click chemistry reaction. These results demonstrated that the obtained microspheres were composed of the target composition. TGA was performed to quantitatively determine the component contents in the composite microspheres. As shown in Figs. 2C , 3 wt% loss of MSP was derived from the content of the PSSMA stabilizer, revealing that the content of magnetic iron oxide accounted for 97 wt%. Upon the encapsulation process with the PAA layer, the weight loss of microspheres increased to 26 wt%. As the stepwise modification, the weight loss of the composite microspheres gradually increased, and the weight loss of MSP@PAA-ADA was approximately 37 wt%. The phenomenon of the gradual increase in weight loss of obtained microspheres with the progress of stepwise modification also illustrated that the successful progress of each step of the reactions. The magnetic properties of the composite microspheres were evaluated using a vibrating sample magnetometer. As depicted in Fig. 2D, the absence of coercivity and remanence confirms the superparamagnetic nature of the microspheres. The saturation magnetization (Ms) of the MSP was about 69 emu/g. With progressive modification, the Ms value decreased incrementally due to the diminishing proportion of the magnetic core. For instance, the Ms value of MSP@PAA-ADA was reduced to 33 emu/g. Despite this reduction, the composite microspheres still meet practical application requirements and can be efficiently separated from solution within 60 s.

### 3.2 The performance of MSP@PAA-ADA in labeling bacteria *in vitro*

To investigate the feasibility of MSP@PAA-ADA for bacterial labeling, we first assessed its bacterial toxicity. Using *F*. *n* as a representative strain, we evaluated the effects of MSP@PAA-ADA at concentrations ranging from 0 to 1.6 mg/mL on bacterial growth activity. As shown in Fig. 3A and Fig. S2, significant reductions in bacterial colony counts were observed at MSP@PAA-ADA concentrations of 0.8 and 1.6 mg/mL. Relative decreases (without statistical significance) occurred at 0.4 and 0.2 mg/mL, while concentrations between 0.00625 and 0.1 mg/mL showed no significant alteration in colony counts. To increase the concentration of MSP@PAA-ADA as much as possible to better label bacteria without affecting bacterial activity, 0.1 mg/mL was selected as the working concentration for subsequent experiments. Following 1-h incubation of salivary microbiota with MSP@PAA-ADA, live/dead staining revealed no distinct differences in SYTO9 and PI fluorescence intensities between treated and untreated groups (Fig. 3B). This visual evidence demonstrates that MSP@PAA-ADA exposure exerts no significant impact on the morphology of proliferating bacteria or cellular viability. Then MSP@PAA-Alkyne and MSP@PAA-ADA were incubated with the saliva sample for 1 h at 37 °C. After that, the mixture was carefully poured into a test tube containing sterile PBS. A magnet was then placed next to the tube to collect the magnetic bacteria. The process is schematized in Fig. 3C. MSP@PAA-Alkyne and MSP@PAA-ADA can be seen to be visibly clustered in proximity to the magnet, with a greater propensity for MSP@PAA-ADA aggregates (Fig. 3D). After the collection of these aggregates, Gram staining was employed to observe that MSP@PAA-ADA exhibited enhanced microorganism adsorption in comparison to the control group. The presence of a diverse array of microbial species, encompassing Gram-positive and Gram-negative *bacilli*, *streptococci*, and *cocci*, was identified (Fig. 3E). CFU assays indicated that MSP@PAA-ADA labeled *F. n* with an efficiency of approximately 88%, while CFU counts showed no significant difference before versus after labeling (Fig. S3). Furthermore, ampicillin bacterial pretreatment, high-concentration free D-amino acid medium supplementation, and bacterial heat inactivation all markedly diminished the binding affinity of MSP@PAA-ADA to target bacterial cells (Fig. S4). The results obtained demonstrate that MSP@PAA-ADA exhibits both effective microorganism adsorption and magnetic properties.

### 3.3 Community structure and species composition of MSP@PAA-ADA-labeled microbiota

**Fig. 4:**
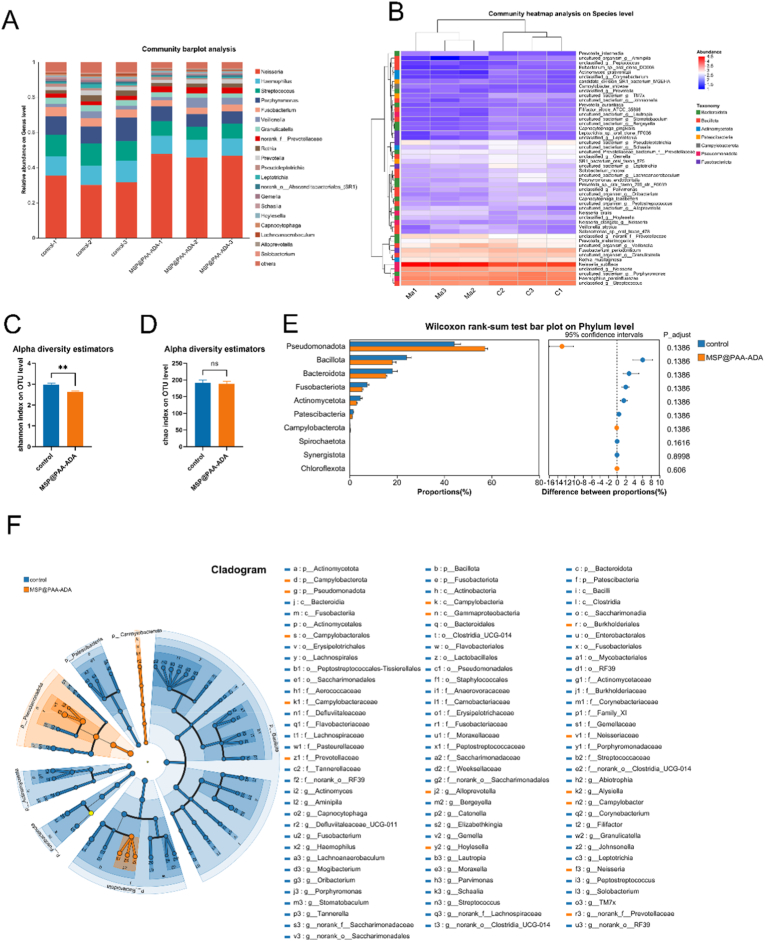
Alterations of the salivary microbial phylotypes associated with MSP@PAA-ADA. (A) Community bar plot analysis showing the top 20 genus with highest abundance and others before (control-1-3) and after (MSP@PAA-ADA-1-3) MSP@PAA-ADA treated. n = 3 per group. (B) Community heatmap analysis showing the top 50 species with highest level before (C1-3) and after (Ma1-3) MSP@PAA-ADA treated. (C) Shannon Index of operational taxonomic unit (OTU) level, ∗∗*p* < 0.01. (D) Chao Index of OUT level. (E) Discriminative species at phylum level were identified using Wilcoxon rank-sum test. (F) A cladogram for taxonomic representation based on LEfSe. Blue indicates enrichment in control group, and orange indicates the taxa enriched in samples after incubation with MSP@PAA-ADA. (For interpretation of the references to color in this figure legend, the reader is referred to the Web version of this article.)

To investigate whether there is a difference in the adsorption capacity of MSP@PAA-ADA for different microorganisms, a microbial diversity assay was performed on the collected oral saliva samples and the microorganisms adsorbed by MSP@PAA-ADA. A total of 422,250 high-quality sequences were produced, and 12 phyla, 18 classes, 37 orders, 58 families, 104 genera, 180 species, and 248 OTUs were identified for the two Groups. The top genera of both groups (including *Neisseria*, *Haemophilus*, *Streptococcus*, *Porphyromonas*, *Fusobacterium*, *Veillonella*, *Granulicatella*, *Prevotellaceae*, *Rothia*, *Prevotella*, *Pseudoleptotrichia*, *Leptotrichia*, *SR1 Absconditabacteriales*, *Gemella*, *Schaalia*, *Hoylesella*, *Capnocytophaga*, *Lachnoanaerobaculum*, *Alloprevotella*, and *Solobacterium*) accounted for >90% of the total genera found in the salivary samples of the control and MSP@PAA-ADA groups and exhibited analogous community species composition (Fig. 4A). The top 50 abundant species in each sample are displayed in the heatmap (Fig. 4B). The community diversity of the microbial community in the MSP@PAA-ADA group was significantly lower than that in the control group (Fig. 4C), but there was no significant difference in community richness (Fig. 4D). The significance level of the difference in phyla abundance was evaluated with Wilcoxon rank-sum test according to the obtained community abundance data, and species with significant variances between groups were obtained at the genus level. There were no significant differences between two groups at the top 10 phyla (Fig. 4E). The most discriminatory taxa between groups from phylum to genus level were further identified using LEFSe with logarithmic LDA score >2.0. The 100 taxa showed differential distribution in the two groups. *Bacillota*, *Pastescibacteria*, *Actinomycetota*, and *Fusobacteriota* at the phyla level, *Flavobacteriales*, *Pseudomonadales*, and *Enterobacterales* at the order level, and *Tannerellaceae*, *Porphyromonadaceae*, and *Burkholderiaceae* at the family level were more prevalent in the control group, while *Campylobacterota*, *Neisseriaceae*, and *Prevotellaceae* were more abundant in the MSP@PAA-ADA group (Fig. 4F).

### 3.4 Dual MSP@PAA-ADA/Cy5ADA labeling for tracking oral microbiota translocation to gut

**Fig. 5:**
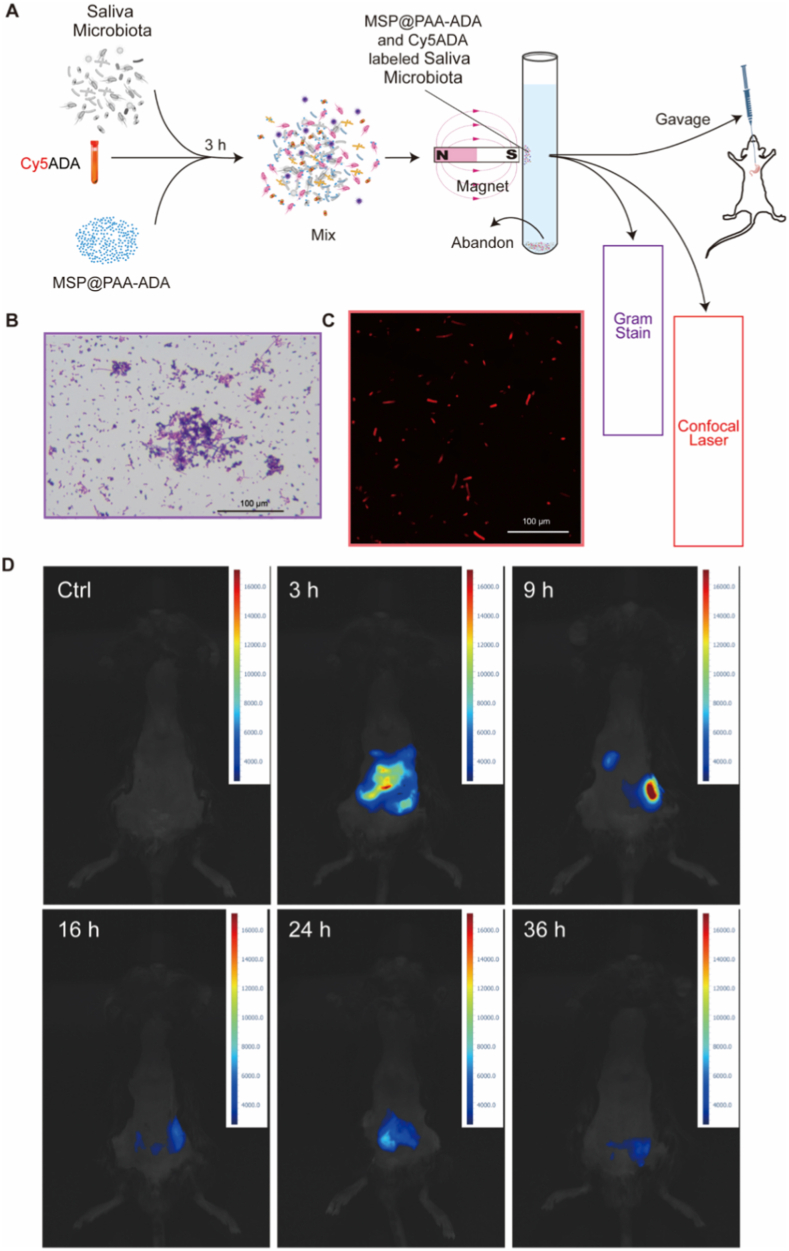
MSP@PAA-ADA and Cy5ADA co-labeling staining strategy. (A) Schematic process of MSP@PAA-ADA and Cy5ADA co-labeling strategy. (B) The gram stanning of microbiota after magnetic separation. Scale bar is 100 μm. (C) The confocal laser microscopic observation showed the magnetically separated microbiota exhibit red fluorescence. Scale bar is 100 μm. (D) The *in vivo* near-infrared images of co-labeled microbiota in mice at 0, 3, 9, 16, 24, and 36 h after gavage. Three mice per time point. (For interpretation of the references to color in this figure legend, the reader is referred to the Web version of this article.)

To understand whether MSP@PAA-ADA affects intestinal translocation of oral strains, Cy5ADA was used in this study for visualization and tracking. Initially, the salivary microbiota, Cy5ADA and MSP@PAA-ADA, were mixed and incubated, after which strains with magnetic properties were screened using magnets and differential centrifugation (Fig. 5A). The strains that were subjected to screening were found to be diverse, containing Gram-positive and Gram-negative *bacilli*, *cocci*, and *streptococci*, as detected by Gram staining (Fig. 5B). Laser confocal scanning microscopy revealed that these strains exhibited red fluorescence, thereby demonstrating that the MSP@PAA-ADA-conjugated strains possessed the capacity to bind to Cy5ADA (Fig. 5C). Subsequently, mice were gavaged with the screened colonies. The distribution and excretion of the strains in mice at different time points post-gavage was observed by *in vivo* near-infrared imaging (Fig. 5D). The imaging data reveal a distinct dynamic pattern of fluorescence signal intensity and distribution over time. At 3 h after administration, the gastrointestinal tract of mice showed a large fluorescent signal, indicating the initial accumulation and distribution of the probe within the digestive system. By 9 h, the fluorescence signal intensity further increased and the distribution centralized. However, at 16 h, the fluorescence signal began to attenuate compared to 9 h, reflecting a portion of the probe starting to be eliminated from the body. Subsequently, at 24 h and 36 h, the fluorescence signal continued to weaken, eventually approaching background levels. To visualize the distribution of the probes in the mouse intestine more intuitively, the mouse gastrointestinal tract was dissected and subjected to near-infrared fluorescence detection, and a time-course line plot of fluorescence intensity changes was generated in parallel (Fig. S5). The results showed an emptying process like that *in vivo*, and the probes were significantly aggregated in the cecum during the 3 h to 36 h process. This demonstrates that the probe excretion in mice is a slow and sustained dynamic process, transitioning from initial distribution and accumulation to gradual metabolism and clearance. The imaging results provide a comprehensive visualization of the MSP@PAA-ADA-conjugated strains *in vivo* behavior, offering valuable insights into its pharmacokinetic properties and interactions within the biological system.

### 3.5 Sequential MSP@PAA-ADA and Cy5ADA labeling reveals the intestinal translocation of viable oral microbiota

**Fig. 6:**
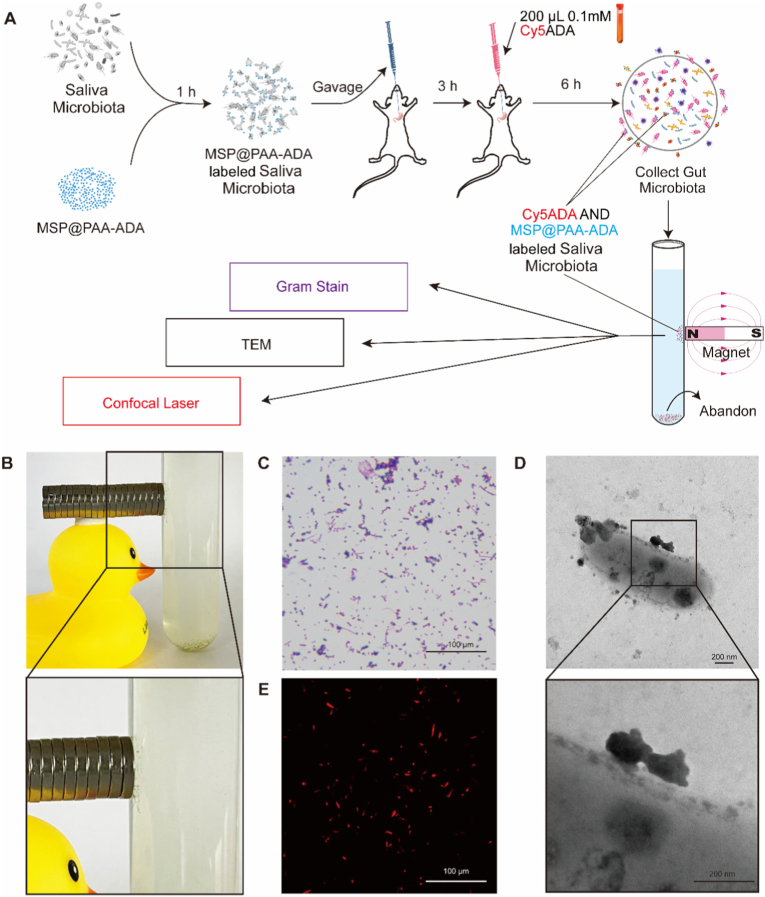
Sequential labeling strategy of MSP@PAA-ADA and Cy5ADA. (A) Schematic steps of MSP@PAA-ADA and Cy5ADA sequential labeling strategy. (B) The magnified view showing the material adsorbed around the magnet. (C) Gram staining, (D) TEM images, and (E) confocal laser scanning microscopy (CLSM) images of the collected gut microbiota after magnetic separation. Scale bar is 100 μm (C, E) and 200 nm (D).

To verify whether the MSP@PAA-ADA-labeled strains could maintain their activity in the gastrointestinal tract, the MSP@PAA-ADA and Cy5ADA sequence labeling strategies were further designed. As shown in Fig. 6A, firstly, the salivary microbiota was incubated with MSP@PAA-ADA for 1 h and then the mice were gavaged, Cy5ADA was instilled after 3 h, and then the contents of the cecum of the mice were collected after 6 h, and then magnetically sorted for the serial assay. Fig. 6B shows magnetically separated strains, aggregated and adsorbed around a magnet. Gram staining showed that the magnetically isolated strains were diverse, containing Gram-positive and Gram-negative *bacilli*, *cocci*, *streptococci*. (Fig. 6C). Transmission electron microscopy showed that MSP@PAA-ADA was adsorbed on the bacterial surface (Fig. 6D). Laser confocal microscopy showed magnetically isolated strains with red fluorescence (Fig. 6E). Comparison of live/dead staining between MSP@PAA-ADA-labeled salivary bacteria before gavage and those recovered from cecum at 9 h post-gavage revealed no statistically significant change in bacterial viability, yet a reduction in bacterial load (Fig. S6). Successful labeling of MSP@PAA-ADA and Cy5ADA was also observed using *F*. *n* as an indicator strain (Fig. S7). These results indicated that the MSP@PAA-ADA-labeled strain was able to maintain the ability to uptake Cy5ADA, which means it was active, after entering the gastrointestinal tract of mice for 3 h.

## 4 Discussion

In this paper, a magneto-fluorescence sequential labeling strategy based on magnetic nanoparticles MSP@PAA-ADA and Cy5ADA fluorescent dyes was constructed to monitor the process and activity of oral-gut microbiota translocation. Evidence suggests that oral bacteria migrate to the gut through swallowing and colonize across various disease states, especially when inflammation or weakened mucosal barriers are present [3,21]. Yet, much of this evidence comes from community-level correlation analysis. This makes it difficult to prove the direction of migration or the metabolic activity of these microbes once they reach the gut. To solve this, our study applies a specific labeling and separation strategy. Utilizing the synergistic effect of MSP@PAA-ADA and Cy5ADA metabolic probes, only the active bacteria are targeted, avoiding interference from dead or free DNA. Based on the combination of magnetic solid-phase extraction and fluorescence labeling, the magneto-fluorescence bimodal labeling strategy can realize the tracing and activity detection of intestinal colonies in a temporally and spatially controllable manner, which provides a brand-new technological means to study the causality between oral-gut microbiota translocation and intestinal inflammation, metabolic disorders, and other diseases. Since this strategy was tested only in mice, the results require careful interpretation before applying them to humans. Differences in gut bacteria and physiology mean that more clinical studies are needed to confirm these initial findings.

Magnetic nanoparticles have been extensively studied in medicine, including magnetically induced thermotherapy, drug delivery, imaging and biosensing applications [22]. The current application modes of magnetic nanoparticles mainly lie in antimicrobial or cell labeling, and there are fewer studies related to bacterial labeling [[23], [24], [25], [26], [27], [28], [29]]. In this study, MSP@PAA-ADA constructed by creatively using D-amino acids as a modified structure can be taken up by bacteria without affecting the bacterial activity. The PAA coatings increase the stability and biocompatibility of the particles and help in bio-adhesion [30]. Magnetic nanoparticles smaller than 200 nm can enter the intracellular space by endocytosis, and the particle size of MSP@PAA-ADA is around 160 nm. SEM imaging reveals that the surface roughening induced by D-amino acid modification increases the effective surface area, which may facilitate bacterial adhesion and may prolong intestinal residence time [31,32]. Transmission electron microscopy (TEM) shows that MSP@PAA-ADA mostly binds to the bacterial surface rather than entering the intracellular space. This is likely due to its hydrophilicity and negatively charged surface, which causes electrostatic repulsion with the similarly negatively charged bacterial cell membrane [33,34]. As shown in Fig. 3A, the reduction in CFU counts at concentrations of 0.8 mg/mL and 1.6 mg/mL may stem from the deep integration of a large amount of MSP@PAA-ADA into the peptidoglycan layer. This process can trigger physical shielding and membrane perturbation. Such a barrier hinders the uptake of nutrients and the discharge of metabolic wastes, thereby inhibiting bacterial proliferation [35]. At the same time, the extensive incorporation of particles may alter the mechanical properties of the cell wall. This can lead to structural stress or induce the production of reactive oxygen species (ROS), which reduces cell viability at high concentrations [[36], [37], [38]]. Besides, MSP@PAA-ADA is magnetic and can be remotely controlled by magnetic field, thus realizing magnetic separation and targeted delivery, which is also the direction of subsequent research.

The 16s sequencing of salivary colonies before and after MSP@PAA-ADA binding revealed that MSP@PAA-ADA was able to bind a wide range of bacteria in a broad manner without affecting the total number of species within the colonies. However, the decrease in shannon index indicates that MSP-PAA-ADA leads to a decrease in the uniformity of species distribution within the colony. Further LEfSe analysis revealed a significant enrichment of *Campylobacterota*, *Neisseriaceae*, and *Prevotellaceae* in the microbiota labeled by MSP@PAA-ADA (Fig. 4F). These enriched taxa possess distinct biological characteristics. *Campylobacterota* is common Gram-negative, spiral-shaped bacteria primarily inhabiting the oral and gastrointestinal mucosa [39]. *Neisseriaceae* is gram-negative anaerobic diplococci and are mostly resident in the respiratory tract [40]. *Prevotellaceae* is a gram-negative anaerobic bacterium found in the human oral cavity, skin, intestinal tract and vagina [41]. The increase in their relative abundance suggests that MSP@PAA-ADA has a higher capture efficiency for these specific taxa. The observed shifts in microbial community structure may result from the selective interaction of MSP@PAA-ADA with bacteria of different activities. Although MSP@PAA-ADA shows no obvious effect on bacterial growth and activity, whether it induces physiological stress that affects bacterial function requires further study, which does not impair its utility for bacterial visualization and separation. The potential impact of non-specific binding during data analysis was also considered. Although MSP@PAA-ADA carries a negative surface charge and primarily labels live bacteria via D-amino acid side chains, a trace amount of non-specific adsorption cannot be entirely ruled out. Consequently, multiple rounds of PBS washing and differential centrifugation were performed to remove most impurities. These steps minimized interference from debris or mucus, ensuring that the sequencing results accurately reflect the overall trends of the microbial community.

Existing studies for imaging magnetic nanoparticles mostly focus on magnetic resonance imaging, which has high spatial resolution and unlimited penetration depth [42]. Fluorescence-activated cell sorting (FACS) is a high-precision technique that isolates individual cells based on their specific physical and fluorescent properties but requires making single-cell suspensions [43]. In contrast, our magnetic platform enables the rapid enrichment of bacteria from large-volume samples using simple hardware, which makes the operation much easier. Recently, many researchers have also used Fluorescent D-amino Acids (FDAAs) for live-cell imaging, as these tools help observe how bacteria grow and divide in different environments [10,44,45]. The magneto-fluorescence sequential labeling strategy innovatively proposed in this study enables real-time monitoring of probe orientation through near-infrared imaging and supports longitudinal tracking within the same animal, thereby enhancing data consistency and better reflecting real biological processes with greater simplicity [[46], [47], [48]]. This strategy is suitable for real-time monitoring of intestinal translocation of specific microbiota, which helps to determine the survival ability and colonization possibility of the microbiota in the intestinal tract, and provides an effective strategy for the study of the intestinal microbiota and the complex biology of the microbiota in the oral-gut axis.

## 5 Conclusion

This study developed a magnetic composite microsphere, MSP@PAA-ADA, functionalized with D-amino acids, enabling the investigation of bacterial migration from the oral cavity to the gut and its underlying mechanisms. The microspheres exhibit excellent water dispersibility, strong magnetic responsiveness, and no cytotoxicity at experimental concentrations. They specifically bind to peptidoglycan in bacterial cell walls, allowing efficient labeling of diverse oral bacteria without altering native salivary microbiota. Combined with the fluorescent probe Cy5ADA, this enables a magneto-fluorescence sequential labeling strategy for enables simultaneous spatial tracking and viability assessment of intestinal translocated oral bacteria. *In vivo*, labeled oral bacteria rapidly distributed throughout the gut within 3 h, and magnetic separation from the cecum confirmed retention of both magnetic and fluorescent signals, indicating real-time capture and monitoring of live oral bacteria. This approach enables high-precision spatiotemporal tracking of oral bacteria in the gut, with enhanced accuracy through dual-modal imaging. The method provides a powerful, non-invasive tool for studying the roles of oral bacteria in systemic health and offers a rapid platform for bacterial monitoring in clinical and research settings.

## CRediT authorship contribution statement

**Meiling Jing:** Data curation, Formal analysis, Investigation, Methodology, Writing – original draft. **Xiuli Wang:** Data curation, Software, Validation, Writing – original draft. **Yaoxia Li:** Investigation, Software. **Leyi He:** Investigation, Software. **Wenduo Tan:** Software, Validation. **Yujie Zhang:** Investigation, Validation. **Yueyi Yang:** Resources, Software, Validation. **Jing Huang:** Investigation, Resources. **Zhengwei Huang:** Funding acquisition, Project administration, Resources. **Changchun Wang:** Methodology, Project administration, Resources, Supervision, Writing – review & editing. **Chenguang Niu:** Conceptualization, Funding acquisition, Methodology, Project administration, Supervision, Writing – review & editing.

## Declaration of competing interest

The authors declare that they have no known competing financial interests or personal relationships that could have appeared to influence the work reported in this paper.

## Data Availability

Data will be made available on request.
