# Supplementary material for: The utilization of a magneto-fluorescence sequential labeling strategy for the visualization and tracking of intestinal translocation of oral microbiota
Source: Mater Today Bio. 2026 Mar 27;38:103062. doi: 10.1016/j.mtbio.2026.103062 (PMC13087794; doi:10.1016/j.mtbio.2026.103062)
Supplement: Multimedia component 1 [file mmc1.docx]

**Supplementary data**

The utilization of a magneto-fluorescence sequential labeling strategy for the visualization and tracking of intestinal translocation of oral microbiota

Meiling Jing ^#,1^, Xiuli Wang ^#,2^, Yaoxia Li ^2^, Leyi He ^2^, Wenduo Tan ^1^, Yujie Zhang ^1^, Yueyi Yang ^1^, Jing Huang ^1^, Zhengwei Huang ^1^, Changchun Wang ^2, *^, ChenGuang Niu ^1, *^

^1^ Department of Endodontics, Shanghai Ninth People’s Hospital, Shanghai Jiao Tong University School of Medicine; College of Stomatology, Shanghai Jiao Tong University, Shanghai, China; National Clinical Research Center for Oral Diseases, National Center for Stomatology; Shanghai Key Laboratory of Stomatology, Shanghai, 200011, China.

^2^ State Key Laboratory of Molecular Engineering of Polymers, Department of Macromolecular Science, and Laboratory of Advanced Materials, Fudan University, Shanghai 200433, China.

The official email addresses:

Meiling Jing: [gjmeiling@sjtu.edu.cn](mailto:gjmeiling@sjtu.edu.cn); Xiuli Wang: wangxiuliyong@163.com; Yaoxia Li: 19307110414@fudan.edu.cn; Leyi He: 22110440014@m.fudan.edu.cn; Wenduo Tan: tanwenduo@sjtu.edu.cn; Yujie Zhang: [zyjfriendly@163.com](mailto:zyjfriendly@163.com); Yueyi Yang: yyYoung@sjtu.edu.cn; Jing Huang: huang_jing711@126.com; Zhengwei Huang: huangzhengwei@shsmu.edu.cn.

*Corresponding author:

Changchun Wang:

Address: Jiangwan Campus, Fudan University, 220 Handan Road, Yangpu District, Shanghai 200438, China

E-mail address: [ccwang@fudan.edu.cn](mailto:ccwang@fudan.edu.cn).

Chenguang Niu:

Address: No. 639 Zhizaoju Road, Shanghai, 200011, China.

E-mail address: niuchg09@alumni.sjtu.edu.cn.


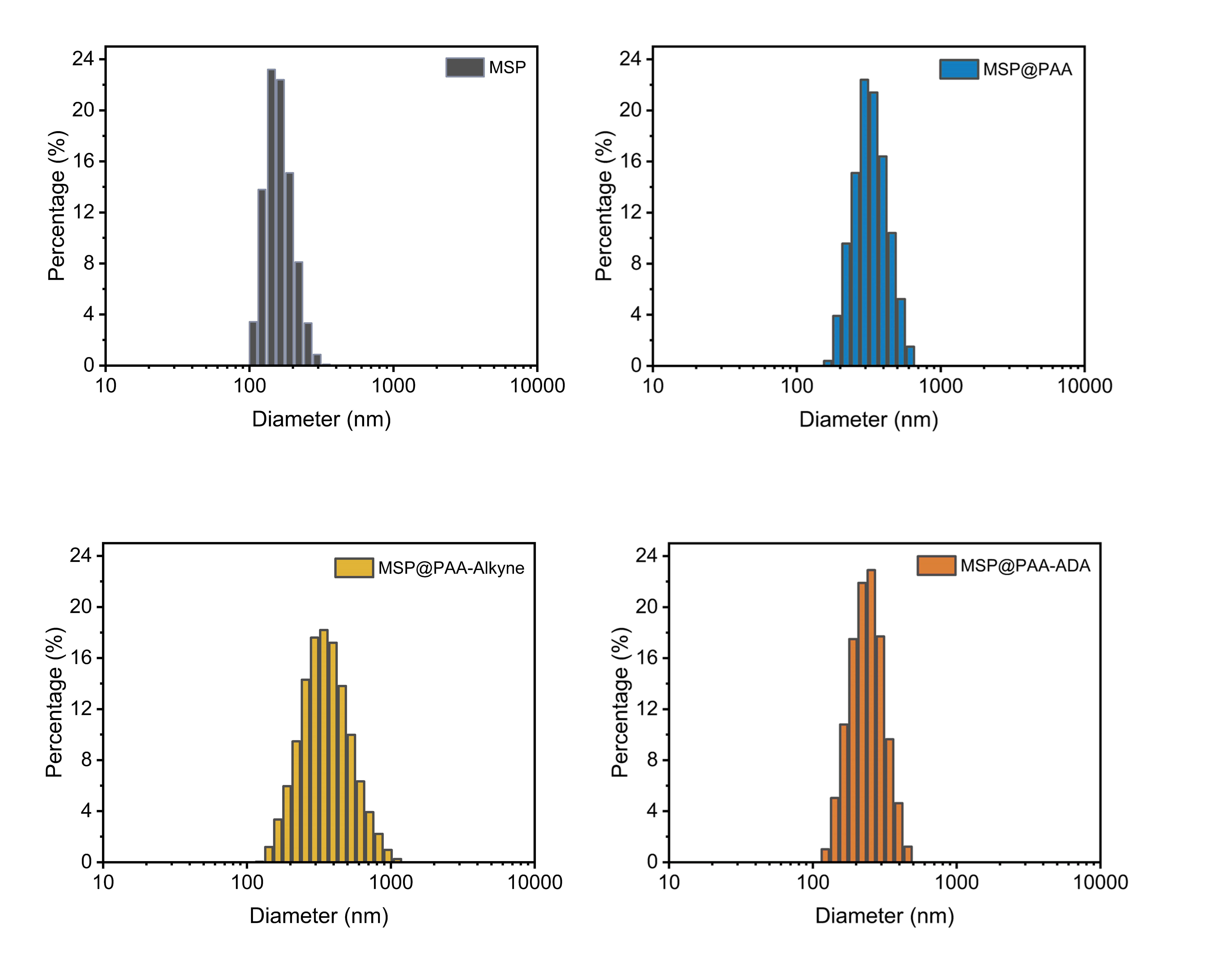


Figure. S1 Size distribution of MSP, MSP@PAA, MSP@PAA-Alkyne, and MSP@PAA-ADA.


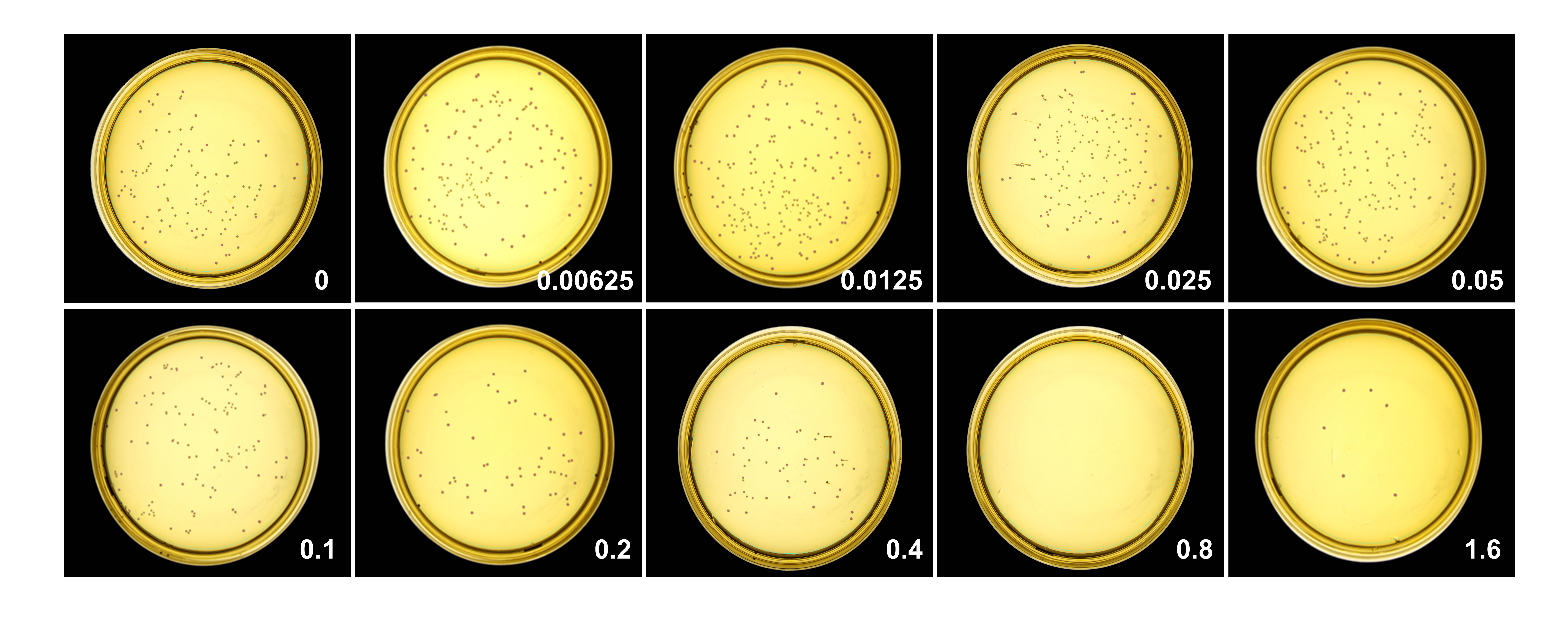


Figure. S2 Representative images of single colony count on BHI media plates after treatment of *F. n* with different concentrations (0, 0.00625, 0.0125, 0.025, 0.05, 0.1, 0.2, 0.4, 0.8, and 1.6 mg/ml) of MSP@PAA-ADA.


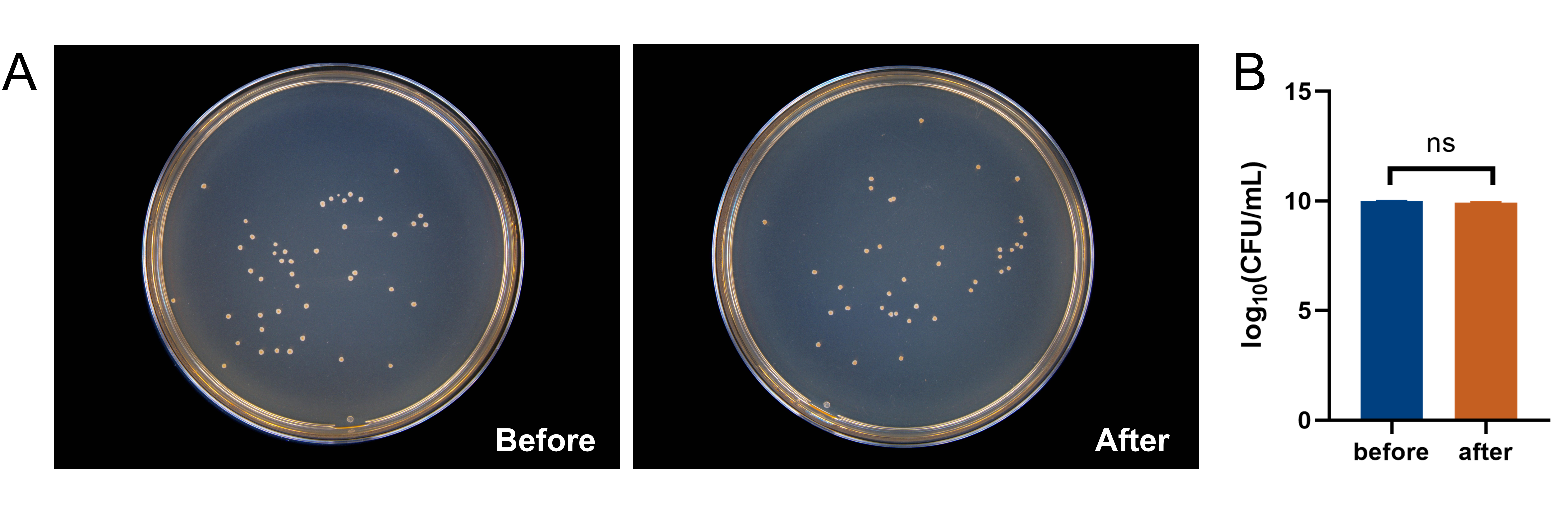


Figure. S3. (A) The colony-counting plates for *F. n* before and after MSP@PAA-ADA labeling and separation. (B) The CFU statistical analysis. *P < 0.05；**P < 0.01；***P < 0.001；****P < 0.0001；ns indicates no statistical siginificance by Student’s t-test.


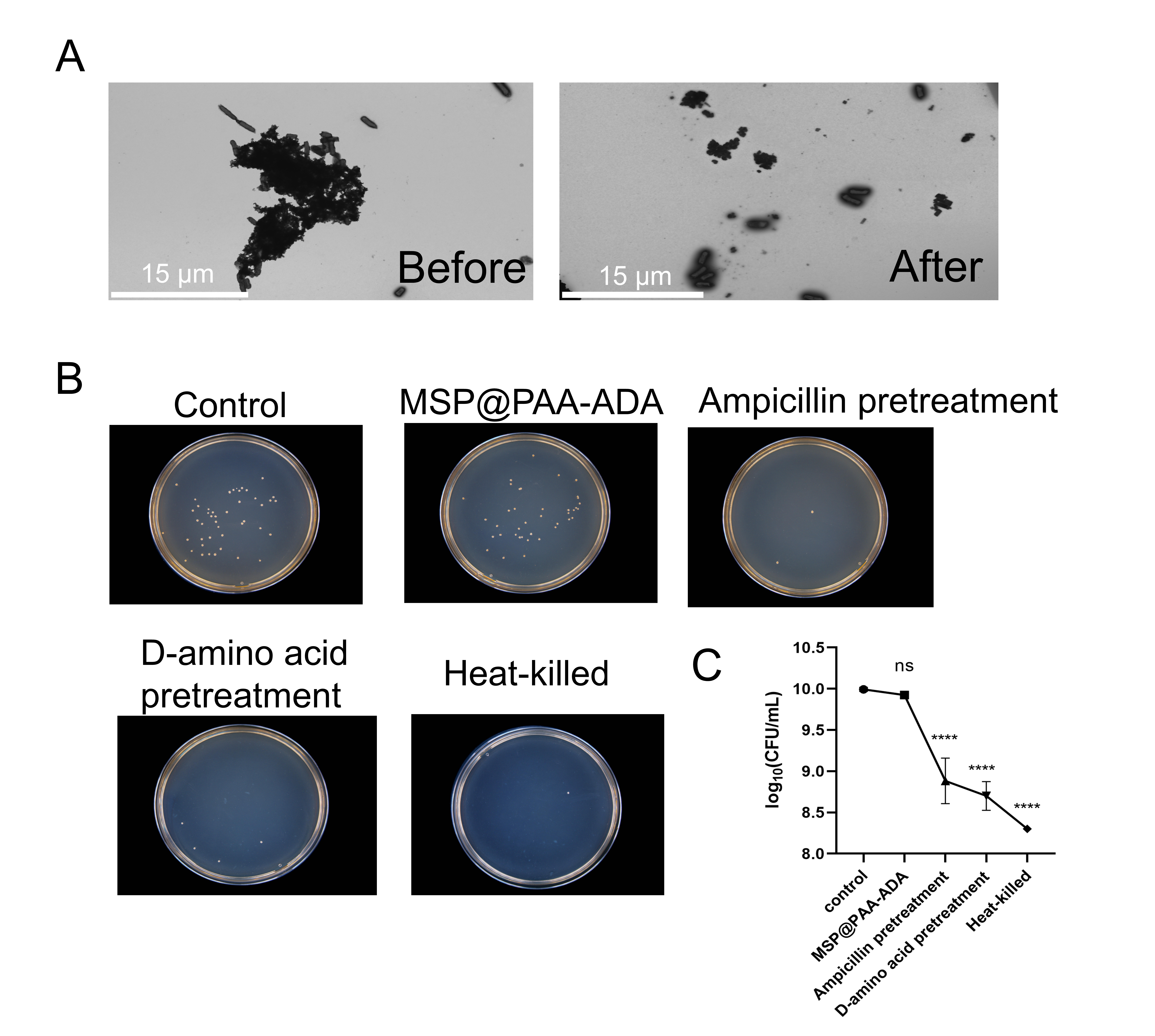


Figure. S4. (A) The TEM images of saliva microbiota before and after ampicillin pretreatment incubated with MSP@PAA-ADA. (B) Representative images of single colony count on BHI media plates of *F. n* pretreated with different situations. (C) Line chart of CFU count statistics. *P < 0.05；**P < 0.01；***P < 0.001；****P < 0.0001；ns indicates no statistical siginificance.


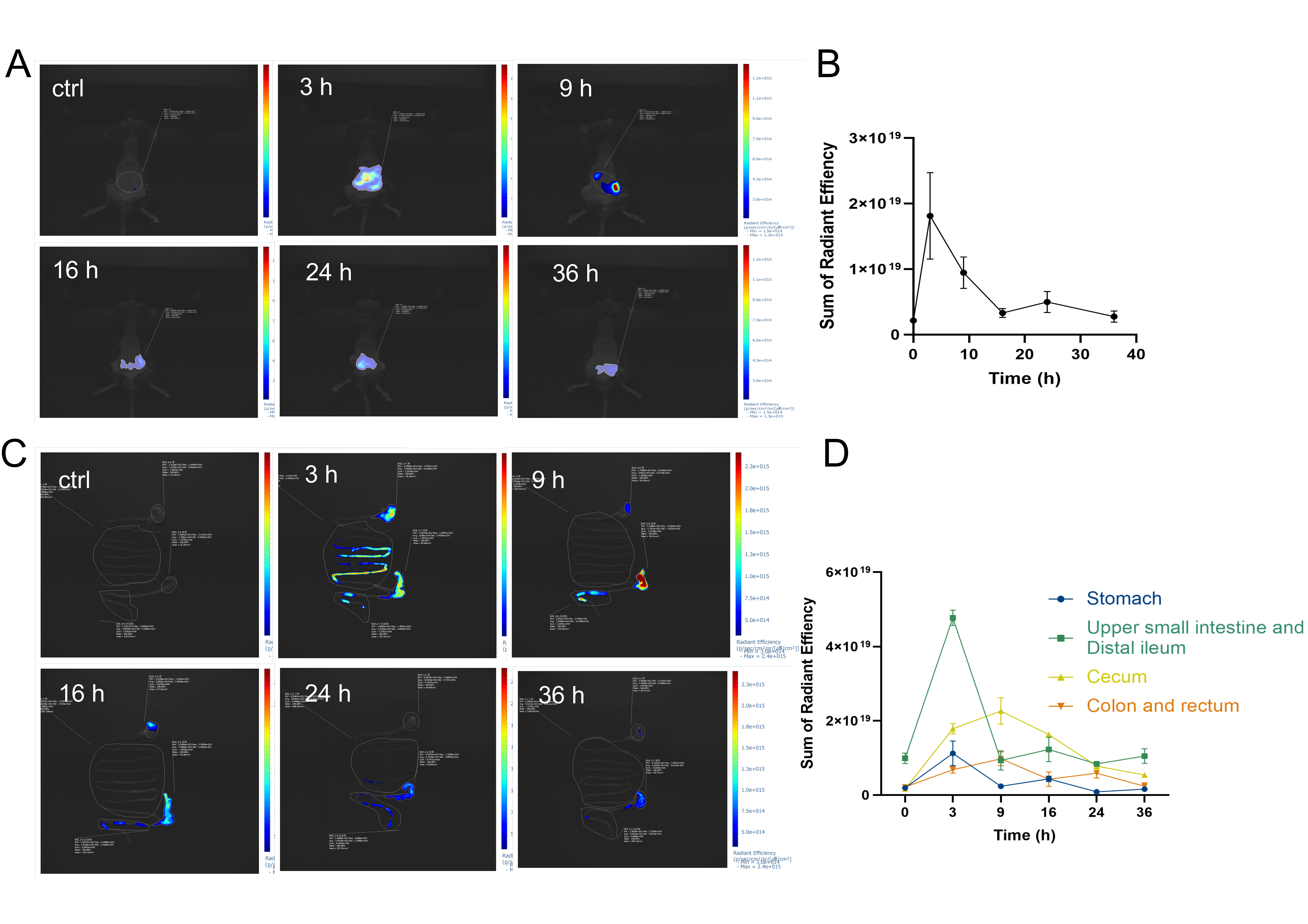


Figure. S5 (A) Representative images of *in* *vivo* fluorescence imaging at 0, 3, 9, 16, 24 and 36 h after gavage. (B) Statistical analysis of the total *in* *vivo* fluorescence value. (C) Representative images of *ex* *vivo* fluorescence imaging at 0, 3, 9, 16, 24 and 36 h after gavage. (D) Statistical analysis of the total *ex* *vivo* fluorescence value.


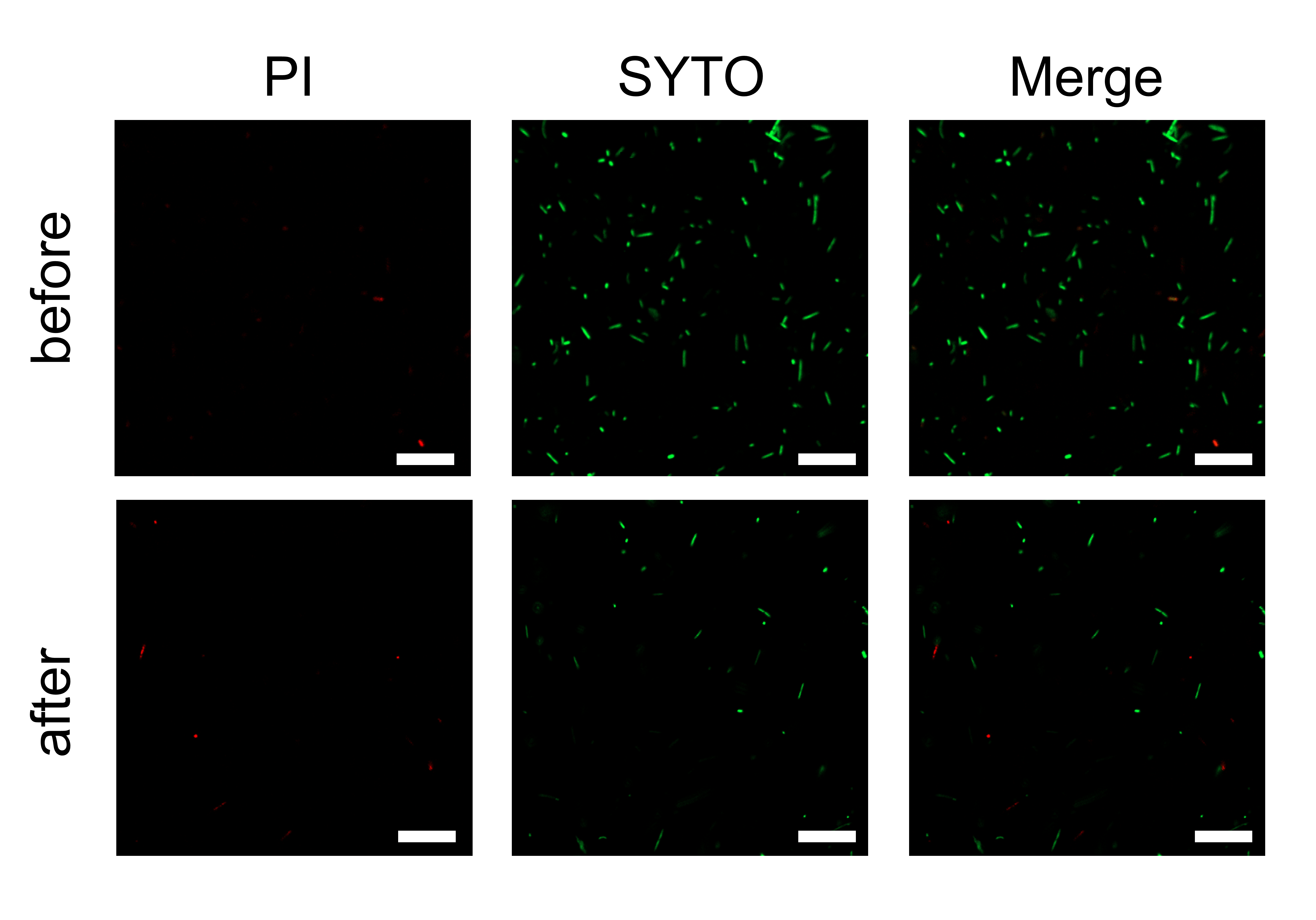


Figure S6. Live bacteria (SYTO 9, Green) and dead bacteria (PI, Red) of salivary microbiota before and after gavage. Scale bar is 20 μm.


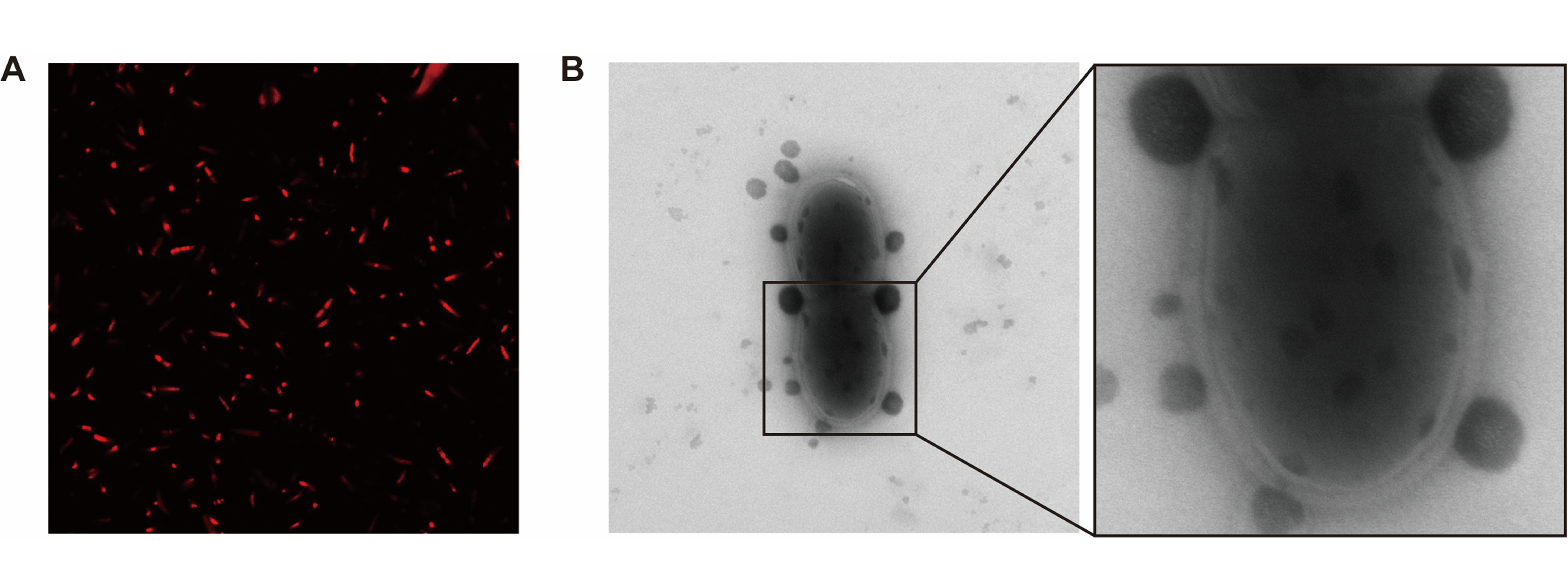


Figure. S7. The representative confocal image (A) and transmission electron microscopy image (B) of *F.* *n* sequential labeled with MSP@PAA-ADA and Cy5ADA.
